# Effects of Growth Stage and Cd Chemical Form on Cd and Zn Accumulation in *Arabidopsis halleri* ssp. *gemmifera*

**DOI:** 10.3390/ijerph18084214

**Published:** 2021-04-16

**Authors:** Hiroshi Kudo, Chihiro Inoue, Kazuki Sugawara

**Affiliations:** 1 Graduate School of Environmental Studies, Tohoku University, Miyagi 980-0845, Japan; hiroshi.kudo.r4@dc.tohoku.ac.jp (H.K.); chihiro.inoue.b1@tohoku.ac.jp (C.I.); 2 Faculty of Science and Technology, Seikei University, 3-3-1 Kichijoji-Kitamachi, Musahino, Tokyo 180-8633, Japan

**Keywords:** *Arabidopsis halleri* ssp. *gemmifera*, cadmium, chemical form, heavy metal accumulation, phytoremediation

## Abstract

Cadmium is a hazardous heavy metal and causes contamination globally. Phytoremediation can potentially become a low-cost and eco-friendly technique for mitigating Cd contamination. *Arabidopsis halleri* ssp. *gemmifera* hyper-accumulates Cd and Zn, and may be used to remediate Cd-contaminated sites. However, few studies have focused on Cd accumulation by *A. halleri* ssp. *gemmifera*. Herein, we demonstrate the accumulation of Cd by *A. halleri* ssp. *gemmifera*. The biomass, Cd, and Zn concentration of the plant increased in the 103 days of experimentation. Cd concentration of soil significantly decreased compared to its initial concentration (≈10%). The material balance of Cd uptake by plant and Cd decrement from soil ranged from 63.3% to 83.7% in each growth stage. Analysis indicated that the water-eluted and exchangeable forms of Cd were stable during the experiment. However, Cd concentration extracted with 0.1 M HCl decreased (25% of initial), and this fraction was not bioavailable. The study exhibits the mass balance of Cd between plant uptake and decrement from the soil and the changes in the chemical form of Cd during stages of *A. halleri* ssp. *gemmifera* cultivation.

## 1. Introduction

Cadmium (Cd) is present in trace amounts in zinc (Zn), lead (Pb), and copper (Cu) ores. During mining and beneficiation of these ores, Cd is discharged into rivers and accumulates in downstream soil through seepage water from mine shafts, drainage, and waste rock deposits [1]. Cd pollution has become a concern in China and other emerging countries as Cd moves through the soil and easily enters the food chain via plant uptake (vegetables, root crops, cereals, and grains) [2,3]. Surveying a wide area covering 70% of China from 2005 to 2013, 8 inorganic pollutants (As, Cd, Co, Cr, Cu, Hg, Mg, and Ni) and three organic pollutants (BHC, DDT, PAH) were discovered in every square km of the surface soil (0–20 cm thick). Soil Cd concentrations were exceeded most frequently, with 7.0% of samples exceeding the standard established by the Chinese Ministry of Environmental Protection [4]. In addition, Tong et al. [5] reported an increased risk of Cd exposure from Chinese urban soils during 2003–2019. Furthermore, wheat, which is consumed by over half of the global population, is known to absorb Cd from its roots and accumulate it in grains. This has increased the concerns regarding potential health hazards [6].

As Cd accumulates in rice, the regions with high rice consumption, such as Japan and other Asian countries, are concerned about the effects of Cd on human health. Although Cd in nature does not cause acute poisoning, chronic poisoning may occur due to long-term Cd exposure at relatively low levels. A serious example of this is Itai-Itai disease, which occurred in the Jinzu River basin in Toyama Prefecture [7]. Itai-Itai disease occurred downstream of Kamioka mine which was the largest Zn mine in the world in the 1960s. A Cd contaminated site was remediated by excavation, which took 33 years in the case of Japan [8]. The cost was approximately 100 million USD for 7.63 km^2^. Generally, for remediation of such contaminated sites, excavation is believed to be a swift and certain remediation technique. However, it is time-consuming, and moving the contaminated soil is expensive. Hence, a low-cost novel technology with a low environmental load is required.

Phytoremediation is an environmental remediation technology that utilizes the biological functions of plants, and the cost and environmental impact are lower than other remediation technologies such as excavation and soil washing [9]. Heavy metals are accumulated in the aerial part of the plant via vital activity, and the plant is then harvested along with the accumulated heavy metals. Plants which can accumulate very high concentration of heavy metals are called hyperaccumulator (>100 mg/kg of Cd). *Arabidopsis halleri* ssp. *gemmifera* is a plant native to East Asia [10] that hyper-accumulates Cd and Zn. In hydroponic cultivation, it accumulated maximum Cd and Zn of 5641 mg/kg dry weight (DW) and 26,400 mg/kg DW, respectively [11,12].

Heavy metals exist in various chemical forms in the soil; water-soluble form is easily eluted by water as ions and is available for plant uptake (i.e., bioavailable). The exchangeable form is bound to layers of clay or adsorbed on the soil surface, and this form can be exchanged for another cation. Therefore, it is considered potentially bioavailable. Carbonate form is bound to carbonates, and oxide form is bound to oxide compounds of Fe and Mn [13]. These heavy metals are eluted in natural conditions, such as weathering, erosion of plant roots, etc. Water-soluble and exchangeable forms are easily eluted by plant and microbial activity (organic acid from roots and microbial activity in rhizosphere) [14]. Potential bioavailability is strongly controlled by the chemical forms of the metals [15].

In the earlier studies with *A. halleri* ssp. *gemmifera*, the total Cd concentration of soil decreased from 5.0 mg/kg to 1.0 mg/kg [16]. Additionally, Cd extracted by 0.1 M HCl (oxide form of Cd) decreased from 3.5 mg/kg to 0.6 mg/kg in the pot experiments [16]. In another experiment, Cd concentration (extracted by 0.1 M HCl) decreased from 3.45 mg/kg to 0.81 mg/kg in the soil after four seasons of extraction by *A. halleri* ssp. *gemmifera* [17]. However, reports do not show a clear material balance between Cd removal from soil and Cd uptake by the plant. Additionally, little is known about behavior of Cd uptake by *A. halleri* ssp. *gemmifera* from soil where only a fraction of metal is bioavailable [18]. Hence, it is necessary to evaluate Cd accumulation by *A. halleri* ssp. *gemmifera*, its material balance between soil and plants and Cd chemical form.

Generally, plants change their nutrient requirements [19] and transcriptional regulation [20] according to growth stage. Although Cd is considered to be non-essential nutrient for *A. halleri* ssp. *gemmifera*, the chemical behavior of Cd resembles that of Zn which is essential nutrient, and it has been reported that Cd and Zn pass through a similar transportation pathway [21]. Therefore, behavior of Cd uptake by plant might be changed according to growth stage. Understanding of detailed behavior of hyperaccumulating plants absorbing heavy metal from early germination to maturity may contribute to improvement of phytoextraction.

The present study investigated Cd accumulation by *A. halleri* ssp. *gemmifera* and the consequent mass balance between soil and plants in a pot experiment. Additionally, the relationship between the chemical form of Cd and its accumulation by plant uptake was explored.

## 2. Materials and Methods

### 2.1. Plant and Soil Preparation and Pot Experiment

Soil containing Cd and Zn was collected from a farmland located downstream of the Hosokura Mine, Tome city, Miyagi Prefecture. The initial concentrations of Cd and Zn in the soil were 4.94 mg/kg and 601.3 mg/kg, respectively. *A. halleri* ssp. *gemmifera* were grown from the seed, and plants with heights of 3 to 5 cm were used for the experiment. The soil was filled in pots of two sizes (small: diameter 11 cm, depth 10 cm; large: diameter 21 cm, depth 20 cm) up to 80% of the total volume and *A. halleri* ssp. *gemmifera* was planted in the center of each pot. The dry weight of the soil in the small pots was 0.61 ± 0.03 kg, and that in the large pots was 3.06 ± 0.19 kg. To ensure that plant growth was not limited by the previous study [22], the amount of soil was kept below 1 g/L plant biomass weight/soil volume. The experiment was conducted from 10 September to 22 December 2014 (103 days). Plants were cultivated under natural conditions until 15 November (66th day), where the maximum temperature in each day was above 15 °C. Thereafter, the plants were moved to a greenhouse maintained at a temperature over 15 °C to ensure stable growing conditions. Plants were harvested at early growth stage (35th and 75th day) from small pots, and at late growth stage (94th and 103rd day) from large pots in triplicates. Sampling was conducted when major changes in plant appearance occurred, with emergence of new leaf after 35 days, death of leaves that were present at planting after 75 days, further leaf emergence after 94 days, and upward leaf elongation after 103 days. During rains, pots were covered with a clear plastic sheet to prevent Cd draining from contaminated soil.

### 2.2. Plants and Soil Analysis Subsection

Plant materials were harvested in a manner to avoid separation of shoots and roots, and then washed with Milli-Q water. Thereafter, plants were divided into shoots and roots and were measured for fresh weight before oven-drying at 60 °C for 48 h, following which the dry-weight was measured. Plant samples weighing 0.01 g were placed in a glass test tube to which 2 mL of HNO_3_ was added and heated at 130 °C by a heat block (Aluminum Block Bath ALB-121, SCINICS) for 2 h. The samples were then digested and diluted by Milli-Q water so that the concentration of the target element was approximately 100 μg/L. The obtained sample was analyzed via inductively coupled plasma mass spectrometry (ICP-MS, ELAN 9000, Perkin-Elmer), just as in Sugawara et al. [23].

After harvesting, soil samples were sieved through a pore diameter of 2 mm and homogenized. Different fractions of the soil sample were extracted by Milli-Q water, (NH_4_)_2_SO_4_, and HCl [24]. The water-soluble fraction was extracted by Milli-Q water and considered to be bioavailable. The exchangeable fraction was extracted by (NH_4_)_2_SO_4_ solution and was also bioavailable because it is assumed that the exudation of the plant dissolves this fraction. HCl-soluble fraction was extracted by HCl solution. The details of extraction are shown in Table 1.

Extracted samples were centrifuged at 3300 rpm (=1500× *g*) for 20 min and the supernatant liquid was diluted by Milli-Q water for ICP-MS analysis. For finding total concentration of Cd and Zn, soil samples were oven-dried at 60 °C until the weights stabilized. The dried soil sample was ground (VIBRATING SAMPLE MILL Tl-100, CMT). The powdered samples were made into pellet using a press machine (Atlas Manual Hydraulic Press 15T, Specac Ltd., Orpington, UK) and then analyzed via X-ray fluorescence spectrometer (XRF, Epsilon 5, PANalytical), as also described in Sugawara et al. [23].

## 3. Results

### 3.1. Biomass Yield and Heavy Metals Concentration of Arabidopsis halleri ssp. gemmifera

**Figure 1:**
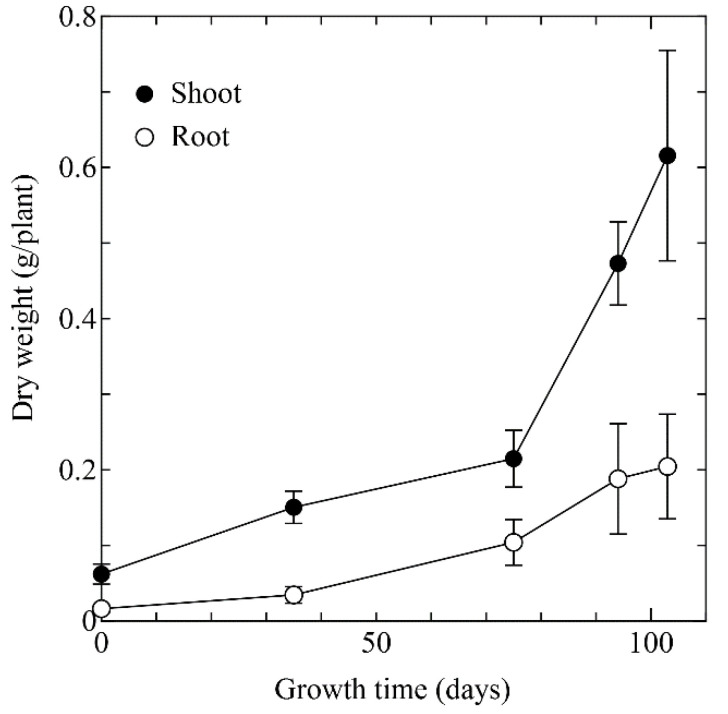
Changes in biomass of shoots and roots of *A. halleri* ssp. *gemmifera.* Black circle: Shoot; white circle: Root. Error bars represent standard deviation of three independent samples.

**Figure 2:**
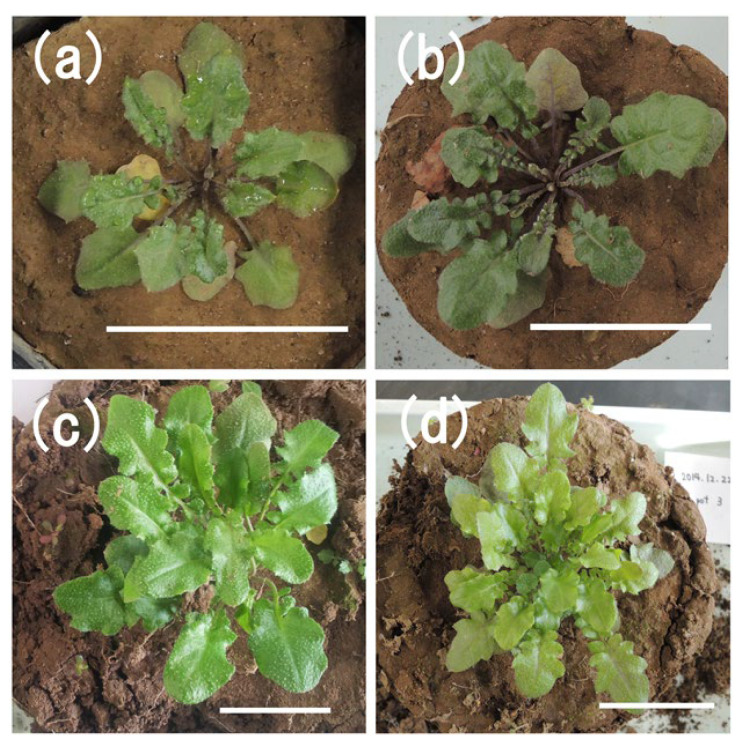
Photo of *A. halleri* ssp. *gemmifera* at (**a**) 35th day, (**b**) 75th day, (**c**) 94th day, and (**d**) 103rd day. Each scale bar is 5 cm.

The plant biomass of *A. halleri* ssp. *gemmifera* is shown in Figure 1. The plant biomass increased well through the experimental period. Specifically, biomass of shoots rapidly increased after the early growth stage (from 75th day to 103rd day). On the 103rd day, biomass of shoots and roots of *A. halleri* ssp. *gemmifera* reached 0.62 ± 0.17 g/plant DW and 0.19 ± 0.06 g/plant DW, respectively. Although the final height of the plant is approximately 30 cm, the stem did not grow vertically in this phase. Therefore, growing old leaves and geminating young leaves allowed an increase in plant biomass (Figure 2).

**Figure 3:**
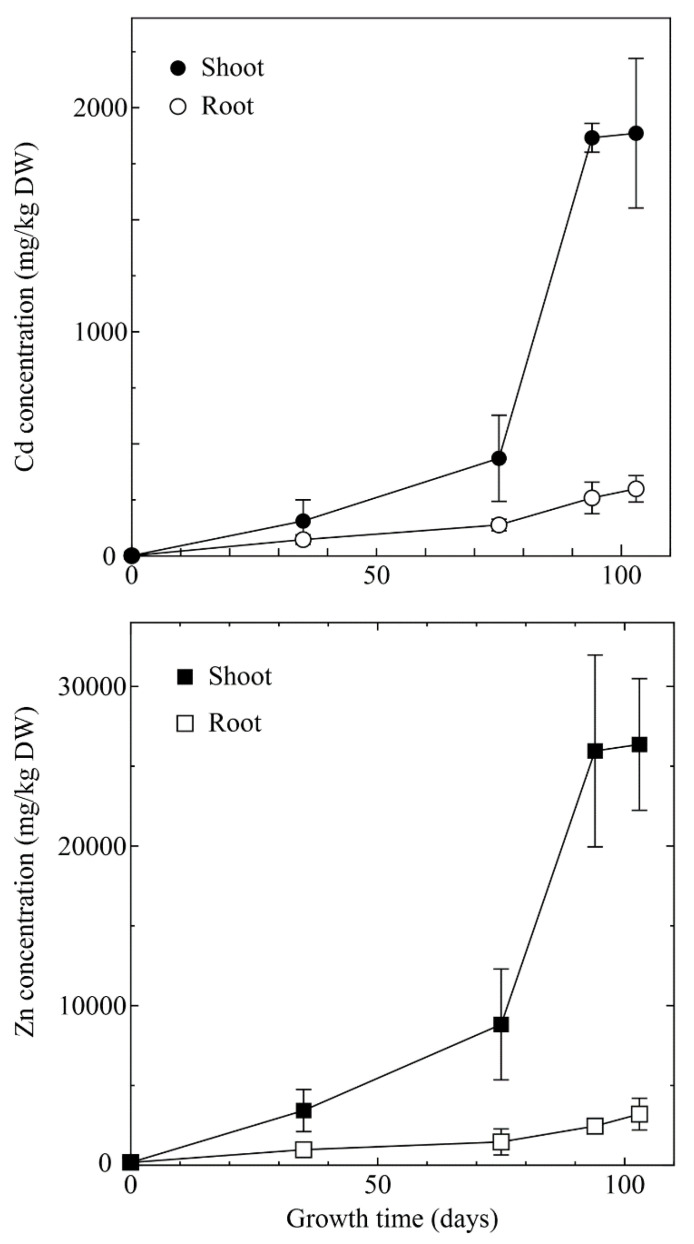
Changes in Cd and Zn concentration in shoots and roots of *A. halleri* ssp. *gemmifera*. Black circle and square: Shoot; white circle and square: Root. Error bars represent the standard deviation of three independent samples.

At the late growth stage, roots were spread across the entire pot area. Hence, it was assumed that plants could absorb heavy metals throughout the soil. The result of Cd and Zn concentration in shoots and roots of *A. halleri* ssp. *gemmifera* is shown in Figure 3. Total heavy metals concentration in shoots rapidly increased after the early growth stage and was the same as the biomass of the shoots. Maximum heavy metal concentrations in shoots on the 103rd day were 1.89 × 10^3^ mg/kg and 2.64 × 10^4^ mg/kg for Cd and Zn, respectively. This result was comparable to that of a previous study performed for 9 months in field conditions using the same Cd and Zn contaminated soil [25]. In this study, well-maintained and suitable growth conditions (regular water supply and well-controlled temperature) were provided, and hence, it was assumed that the plants constantly absorbed heavy metals. Meanwhile, heavy metal concentrations in roots increased moderately. Maximum heavy metal concentrations in roots were 3.00 × 10^2^ mg/kg and 3.20 × 10^3^ mg/kg for Cd and Zn, respectively, on the 103rd day.

### 3.2. Cd and Zn Concentration of Contaminated Soil

**Figure 4:**
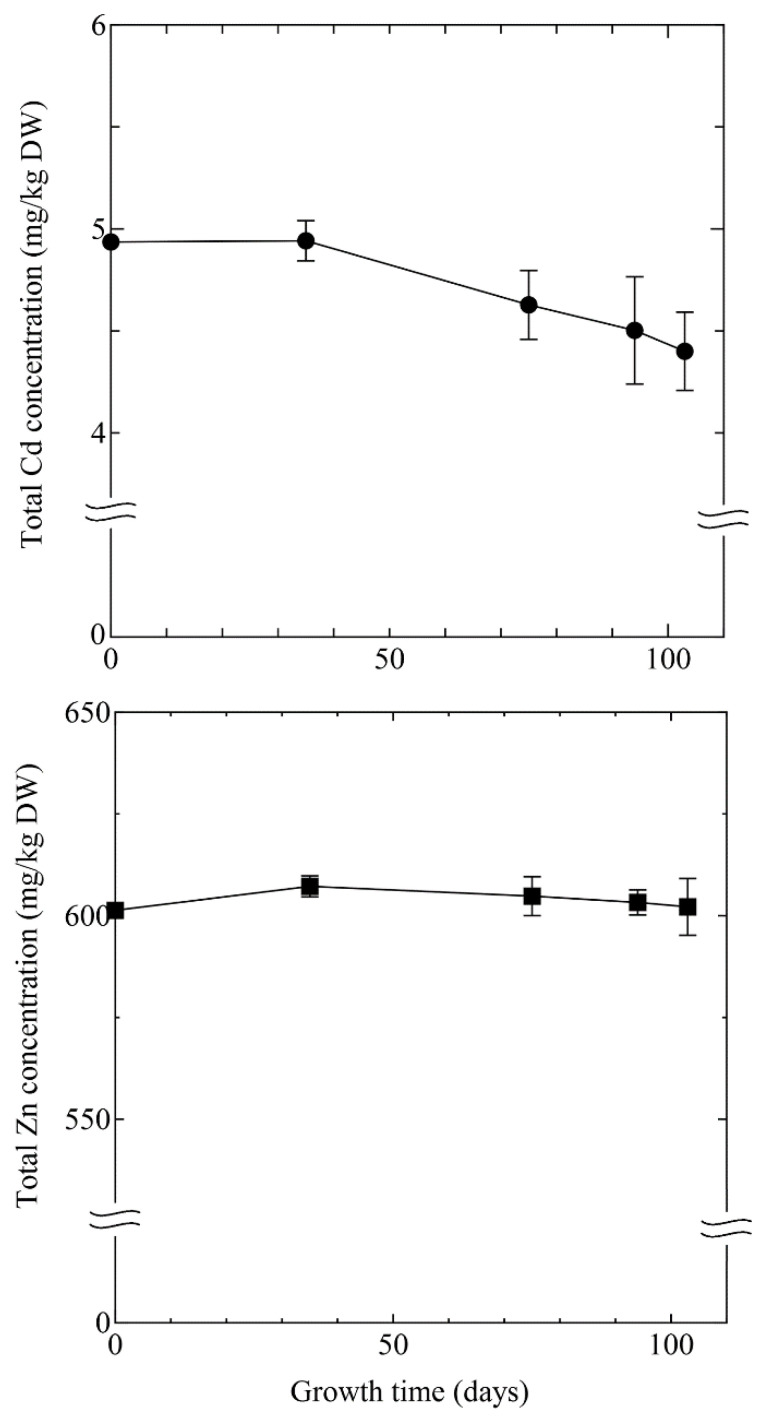
Changes in Cd and Zn concentration in soil. Black circle: Cd concentration; black square: Zn concentration. Error bars represent the standard deviation of three independent samples.

The results of Cd and Zn concentrations of contaminated soil are shown in Figure 4. Total Cd concentration gradually decreased from 4.94 mg/kg to 4.45 ± 0.17 mg/kg. During the experiment, the total Cd concentration reduced by approximately 10%. The results show that the total Cd concentration in soil significantly decreased in only 3 months by *A. halleri* ssp. *gemmifera* cultivation. Contrastingly, the total Zn concentration in soil did not decrease in the experimental period. Although *A. halleri* ssp. *gemmifera* was considered to absorb Zn in the soil (Figure 3), the amount of Zn was large, and hence, Zn uptake by the plant did not reflect the decrease in Zn in soil. Variation in total Zn concentration in the soil during the experiment was observed, which could be attributed to insufficient soil homogenization when adding soil to the pots.

### 3.3. Material Balance between Soil and Plant

The above results show a significant accumulation of Cd by the plants and its consequent reduction from the soil. The ratio of removal (R_p/s_), which represents the mass balance of Cd between soil and plant, was calculated from the results as shown in Section 3.1 and Section 3.2 to reveal the mass balance relationship. Additionally, the translocation factor (TF), bioconcentration factor (BCF), and accumulation factor (AF) were calculated to verify phytoextraction efficiency [26]. TF, BCF, and AF are the indicators of translocation from roots to shoots, accumulation from soil to shoots, and accumulation from soil to the entire plant, respectively. The value of R_p/s_, TF, BCF, and AF are shown in Table 2.

After the 75th day, the mass balance (Cd R_p/s_) ranged from 63.3 ± 16.7% to 83.7 ± 24.3%. This indicated that the most of Cd in soil was absorbed by *A. halleri* ssp. *gemmifera*, rather than drained from the pot. This study highlights the relationship between Cd in plants and in soil, which has not been reported previously.

Cd TF values ranged from 2.03 ± 0.75 to 7.49 ± 1.56. Meanwhile, BCF and AF values rapidly increased from the 75th to the 94th day, and ranged from 31.2 ± 18.1 to 424 ± 80.3 and 28.0 ± 15.3 to 336 ± 59.6, respectively. The earlier study of *A. halleri* ssp. *gemmifera* where AF value of Cd was between 52 and 200 (average = 88) [16]. The AF value in this experiment was higher than that of the earlier study. The Zn TF values ranged from 3.54 ± 1.01 to 10.9 ± 3.87, and were higher than Cd TF values for each sampling time. The BCF and AF values ranged from 5.65 ± 2.17 to 43.8 ± 7.36 and 4.88 ± 1.78 to 34.3 ± 4.59, respectively.

Additionally, it appeared that the translocation speed of heavy metals from roots to shoots was different between the early growth stage and late growth stage in this study (Figure 3). The TF results show that Cd from the root was not transported to the shoot immediately in the early growth stage. Most recently, Qian et al. have observed that Zn is transported from the roots to the aerial parts at a faster rate than Cd, in whole plant of living *A. halleri* ssp. *gemmifera* using radioactive Zn and Cd (personal communication). Therefore, the same trend was confirmed in the present study.

**Figure 5:**
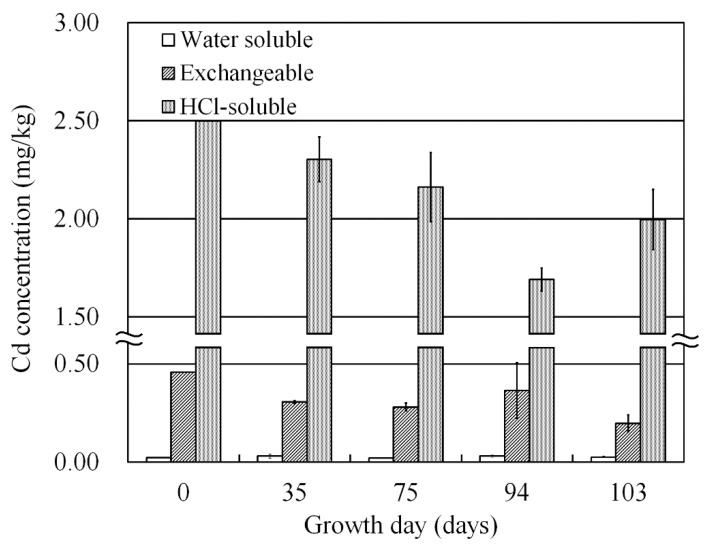
Cd concentration of water-soluble, exchangeable, and HCl- soluble fraction in contaminated soil. White bar: water-soluble fraction; dark gray bar: exchangeable fraction; light gray bar: HCl-soluble fraction. Error bars represent standard deviation of three independent samples.

Extraction experiments were conducted to verify the Cd fraction absorbed mainly by the plant. Cd concentrations of water-soluble fraction, exchangeable fraction, and HCl-soluble fraction in the soil are shown in Figure 5. Cd concentration of water-soluble fraction ranged from 0.020 mg/kg to 0.030 mg/kg in the experimental period, and this fraction was presumed as a bioavailable fraction. Contrastingly, the exchangeable fraction, which was also presumed as a bioavailable fraction, tended to decline. The final concentration was 0.20 ± 0.04 mg/kg, which was half the initial concentration (0.46 mg/kg). The concentration of the HCl-soluble fraction is not bioavailable. However, the concentration slightly decreased from 2.5 mg/kg to 2.0 ± 0.15 mg/kg. The concentration of Cd in the HCl-soluble fraction increased from 94 to 103 days. However, as approximately half of the total concentration of Cd in soil was not leached by HCl, the change to the leachable form possibly occurred because of a change in chemical equilibrium due to absorption of soluble Cd by plants.

## 4. Discussion

Fukuda et al. [21] suggested that Cd and Zn followed a similar pathway because the correlation of the distribution of Cd and Zn between trichomes and leaf was positive on XRF intensities experiment. Bert et al. [27] showed evidence of a generic correlation between Cd and Zn accumulations in the aerial parts of *A. halleri*. Generally, Cd contaminated soil contains Zn, as Cd is derived from zinc ore. *A. halleri* ssp. *gemmifera* is also a hyperaccumulator of Zn. Therefore, phytoextraction of Cd should be performed without interference by Zn. If the Cd pathway is the same as Zn, then Cd uptake by the plant could be obstructed by Zn uptake. However, *A. halleri* ssp. *gemmifera* absorbed Cd adequately (maximum concentration in shoot: 1.89 × 10^3^ Cd mg/kg) without Zn obstruction in this experiment. Hence, *A. halleri* ssp. *gemmifera* can be applied to Cd phytoremediation of soil containing Zn. Furthermore, Cd uptake pathway may be different from that of the Zn uptake. Zn concentration in soil was 100 times that of Cd, and the concentration in shoots was approximately 10 times that of Cd. Although heavy metal concentration in soil was drastically different, the difference in concentration in shoots was smaller than in soil. This result indicated different pathways for Cd and Zn uptake. In cases of iron (Fe) deficiency, the root of *Arabidopsis* induces the expression of the divalent cation transporter such as IRT1, which is essential for the uptake of Fe from the soil and responsible for the uptake of heavy metals such as Cd [28].

In a previous pot experiment conducted for 3–4 months of plant cultivation using 5.4 mg/kg Cd contaminated soil and 3000 cm^3^ pot, the maximum Cd concentration in the plant was 753 mg/kg at the flowering phase [29]. The result obtained in this study was 2.5 times higher than in the previous report. It may represent the difference in chemical and physical properties of soil, such as cation exchangeable capacity (CEC), amount of organic matter, and content of silt influencing Cd mobility. Therefore, it is assumed that the soil properties of this experiment may affect the suitability for Cd uptake by *A. halleri* ssp. *gemmifera*. Contrastingly, soil properties affect plant biomass owing to the presence of nutrients and water retention potential. As the phytoextraction potential is determined by the production of biomass [30], soil properties should be considered in future studies.

**Figure 6:**
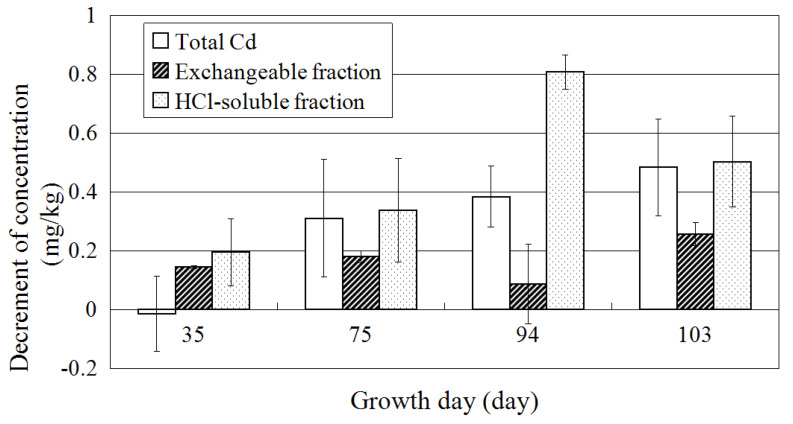
Comparison with decrement of total Cd, exchangeable fraction, and HCl-soluble fraction. White bar: Total Cd; shaded bar: exchangeable fraction; dotted bar: HCl-soluble fraction. Error bars represent standard deviation of three independent samples.

The concentrations of exchangeable fraction and HCl-soluble fraction decreased through the experimental period of the study. Hence, to confirm that the exchangeable fraction is the dominant form absorbed by plants, the difference in the decrement in concentrations between three fractions, namely total Cd, exchangeable, and HCl-soluble, is shown in Figure 6.

Although it is assumed that plants absorb the exchangeable fraction of Cd, decrement in the exchangeable fraction was less than total Cd after 75 days. In contrast, the decrement in the HCl-soluble fraction of Cd was the same as the total Cd on the 75th and 103rd days. Nevertheless, the HCl fraction was not bioavailable. Kubota et al. [16] reported that HCl-soluble fraction (extracted by 0.1 M HCl) in soil decreased for 10 months of *A. halleri* ssp. *gemmifera* cultivation. Degryse et al. [31] suggested that insoluble Cd was slowly released over a long time when exchangeable Cd was depleted. Results of this study indicate that HCl-soluble fraction supplied Cd to an exchangeable fraction when plants absorbed exchangeable Cd. Meanwhile, the chemical form of heavy metals in soil was affected by other factors, such as weathering, activity of microbes, and effect of root exudates. Gradd [32] reported that micro-organisms could mobilize metals through the microbial processes, such as autotrophic and heterotrophic leaching and chelation by microbial metabolites (organic acids) and siderophores. Furthermore, water-soluble and exchangeable fractions of heavy metals increased by the influence of air-drying [33].

The extraction method may give useful information on heavy metals, although the chemical form of Cd in soil is affected by several factors. Some previous reports attempted to upregulate the change in heavy metal chemical forms from stable to bioavailable form [34]. This study clarified for the first time the dynamics of Cd form shifting through plant accumulation. Demonstrating the form of Cd absorbed by the plant and elucidating mechanisms of plant uptake of Cd can improve phytoremediation, and hence investigations with other methods are necessary.

Generally, plants acquire nutrients including metal form soil by releasing root exudates [35] and microorganisms in rhizosphere [36]. In the present study, plant biomass was rapidly increased from the early growth stage to the late growth stage. Cd accumulation in shoot was also increased along with increasing biomass, although *A. halleri* ssp. *gemmifera* reached to maximum Cd concentration in shoot in a short period (3 weeks) regardless of biomass in the previous study using hydroponic culture [11]. This result indicates that the amount of root exudate is increased along with increasing biomass, and it leads to increasing Cd bioavailability in soil. It has been reported that rice root and shoot biomass were positively correlated with root carbon exudation [37]. Additionally, plant growth stage might affect composition of root exudates. Gransee and Wittenmayer [38] reported that the relative amount of sugars in root exudates decreased during plant development. Accordingly, exudates from plant roots might be a remarkable topic for future research. Hoffland [39] reported that rapeseed released citric and malic acids from its root in phosphorus-deficient conditions. Acidification of the rhizosphere enhances the mobilization of rock of phosphate. White lupin [40], maize [41], and soybean [42] also release organic acid to acidify the rhizosphere. Releasing exudates is a common strategy by plants to acquire nutrients. Additionally, wheat exudates enhanced mobility of heavy metals (Zn, Cu, and Cd) in Fe deficient condition [39]. Root exudates of *Nicotiana tabacum* L., which is one of the Cd hyperaccumulators, enhances the mobility of Cd in soil [43]. As organic acid can supply both protons and metal complexing anions, it is assumed that organic acid is heavily involved with Cd mobilization. Contrarily, it was reported that exudates of *Thlaspi caerulescens* known for Cd and Zn hyperaccumulation did not enhance Cd mobility [44]. Thus, the functions of root exudates are different for each plant species and have not been elucidated yet. As it is not clear whether *A. halleri* ssp. *gemmifera* enhances Cd mobility in soil, the relationship between plant uptake and form of Cd must be demonstrated to understand rhizospheric interaction. To summarize, the study revealed Cd accumulation by *A. halleri* ssp. *gemmifera* and its mass balance between Cd chemical form in soil and subsequent shifting in form by plant uptake in pot experiments. This report may contribute to the improvement in the phytoremediation technique and understanding the relationship between heavy metals and plants in the environment. For future research, the relationship between root exudates and Cd fraction in the soil must be investigated.

## 5. Conclusions

In the present study, we investigated the effect of growth stage and chemical form of heavy metal to the accumulation of Cd and Zn by *A. halleri* ssp. *gemmifera*. The biomass, Cd, and Zn concentration of the plant increased in the 103 days of our experiment. Cd concentration of soil significantly decreased compared to its initial (≈10%). Mass balance of Cd uptake by plant and Cd decrement from soil ranged from 63.3% to 83.7% in each growth stage. Analysis indicated that the water-eluted and exchangeable forms of Cd were stable during the experiment. However, Cd concentration extracted with 0.1 M HCl decreased (by 25% of the initial value), and this fraction was not bioavailable. The study showed the mass balance of Cd between plant uptake and decrement from the soil and the changes in the chemical form of Cd during different stages of *A. halleri* ssp. *gemmifera* cultivation. However, influence of *A. halleri* ssp. *gemmifera* on the Cd chemical form change are not investigated adequately. Therefore, in future study, the effect of root exudates of each growth stage on soil needs to be investigated. This study may contribute molecular biological analysis and other methods to facilitate the understanding of the mechanism of hyperaccumulation and tolerance to heavy metals, which is not exhibited by ordinary plants.

## Figures and Tables

**Table 1:** Method of extraction.

Method of Extraction	Solvent	Soil Weight	Solvent Volume	Solvent Concentration	Shaking Speed	Shaking Time
Water extraction *	Milli-Q water	3 g	30 mL	-	200 rpm	6 h
Exchangeable extraction	(NH_4_)_2_SO_4_	1 g	20 mL	0.05 mol/L	200 rpm	4 h
HCl-soluble extraction *	HCl	5 g	25 mL	1.0 mol/L	100 rpm	30 min

* Water extraction and HCl extraction were based on the elution test and measuring content test, respectively, established by Ministry of the Environment in Japan.

**Table 2:** Material balance between soil and plant.

Growth Time (Days)	35 Days		75 Days		94 Days		103 Days	
Cd decrement in soil (mg)	−0.01	±0.08	0.18	±0.11	1.17	±0.33	1.47	±0.39
Cd increment in plant (mg)	0.03	±0.08	0.11	±0.06	0.93	±0.08	1.20	±0.27
Cd Rp/s (%)	17.6	±58.5	63.3	±16.7	83.7	±24.3	83.2	±10.1
Cd TF	2.03	±0.75	3.04	±0.92	7.49	±1.56	6.32	±0.28
Cd BCF	31.2	±18.1	95.2	±44.1	410	±19.1	424	±80.3
Cd AF	28.0	±15.3	72.7	±29.9	311	±19.9	336	±59.6
Zn TF	3.54	±1.01	6.98	±4.17	10.9	±3.87	8.65	±2.14
Zn BCF	5.65	±2.17	14.6	±5.84	43.1	±10.2	43.8	±7.36
Zn AF	4.88	±1.78	10.5	±3.89	32.5	±9.77	34.3	±4.59

Each condition has three replicates. Rp/s of Zn was not calculated because the concentration of Zn in soil barely decreased. TF = Cd or Zn concentration in shoots/Cd or Zn concentration in roots. BCF = Cd or Zn concentration in shoots/Cd or Zn concentration in soil. AF = Cd or Zn concentration in the entire plant/Cd or Zn concentration in soil.

## Data Availability

The data presented in this study are available on request from the corresponding author upon reasonable request.
